# Supplementary material for: Behavior‐ and Cell Type‐Specific Cortico‐Striatal Decoupling in a Parkinson's Disease‐Like Mouse Model
Source: Adv Sci (Weinh). 2025 Dec 23;13(10):e13670. doi: 10.1002/advs.202513670 (PMC12915231; doi:10.1002/advs.202513670)
Supplement: Supplementary file 1 — Supporting File: advs73434‐sup‐0001‐SuppMat.pdf. [file ADVS-13-e13670-s001.pdf]

Supplementary Figures and Legends

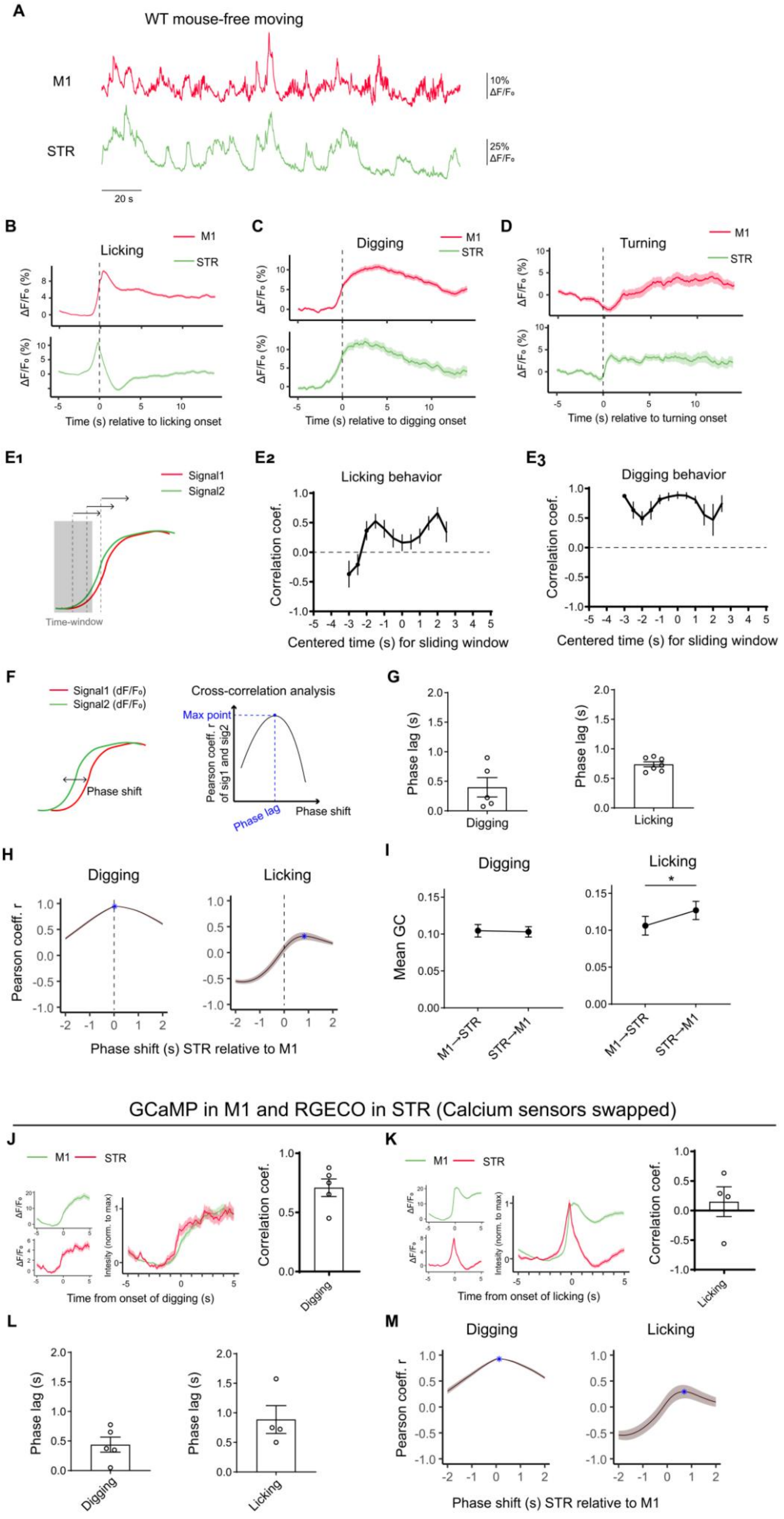

Figure S1. Cortico-striatal coupling was behavior-specific and independent of calcium sensors.

A. Example temporally aligned Ca^{2+} activities recorded from M1 (red) and STR (green) from a wild-type mouse free moving in a lidless cage.

B-D. Prolonged time-window recordings of calcium activity in M1 and striatum (STR).

(Left panel) Temporally aligned calcium traces ($\Delta F/F_0$) for M1 and STR across contralateral turning, licking, and digging behaviors in wild-type mice.

E. Time-resolved cross-correlation analysis of M1 and striatal Ca^{2+} activities using a 4-second sliding window. (E_1): Schematic depiction of analysis. (E_2): Analysis for licking. (E_3): Analysis for digging behavior.

F. Schematic diagram illustrating how the phase lag was calculated.

G. Phase lag (STR relative to M1) of digging or licking calcium signal from wild-type mice. Each point represents a single mouse.

H. Cross-correlation lag distributions diagram of digging or licking-related calcium signal from wild-type mice.

I. Mean GC (granger causality) value per mouse based on digging (left) or licking (right) behavior-related M1 and STR calcium signal, from trails with significant granger causality. Wilcoxon matched-pairs test. *: $P<0.05$.

J-K Normalized $\Delta F/F_0$ traces of M1 (green) and STR (red) temporally aligned to digging-associated (J) or licking-associated (K) behaviors, along with the corresponding correlation coefficient, from wild-type mice injected with region-reversed calcium indicators. Each point represents a single mouse.

L. Phase lag (STR relative to M1) of digging or licking calcium signal from wild-type mice injected with region-reversed calcium indicators. Each point represents a single mouse.

M. Cross-correlation lag distributions diagram of digging or licking calcium signal from wild-type mice injected with region-reversed calcium indicators.

Data were presented as mean ± SEM.

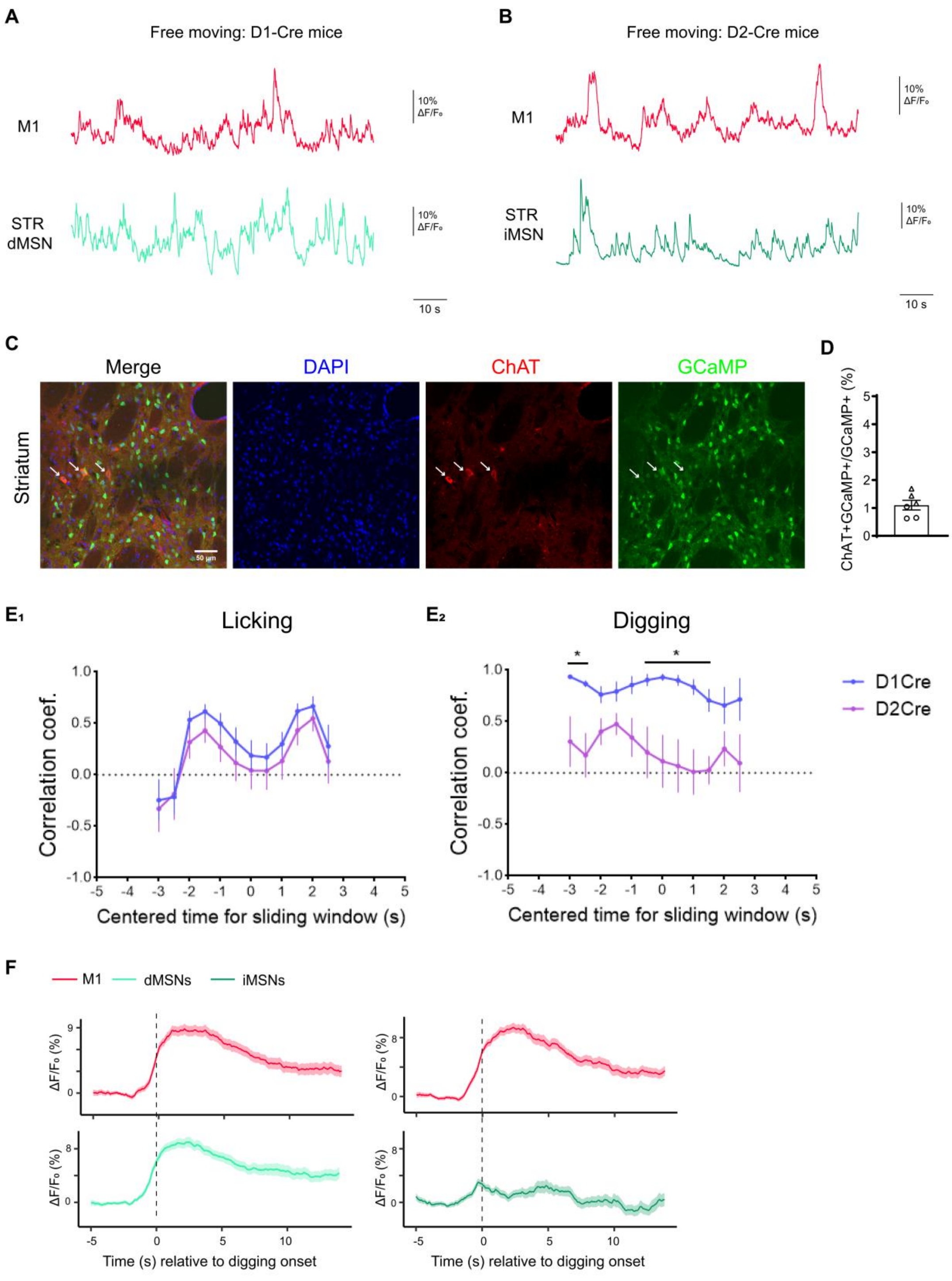

Figure S2. Validation of cell type-specific resolution in the methodology. Related to Fig2.

A. Example temporally aligned Ca^{2+} activities recorded from M1 (red) and STR dMSNs (aqua) from a D1R-Cre mouse during free moving.

B. Example temporally aligned Ca^{2+} activities recorded from M1 (red) and STR iMSNs (dark green) from a D2R-Cre mouse during free moving.

C-D. Co-immunostaining of ChAT in coronal brain slices harvested from D2-cre mice

expressing DIO-GCaMP in the striatum. (C) Representative images. Scale bar: 50 μm. (D) Quantification of the percentage of $ChAT^+$/$GCaMP^+$ cells relative to all $GCaMP^+$ cells (6 views from 2 mice).

E. Comparison of correlation coefficient of M1 and striatal Ca^{2+} activities within sliding window (length: 4s) from D1-Cre mice (blue) and D2-Cre mice (purple) for licking (E_1, D1: n = 7; D2: n = 8) and digging behavior (E_2, D1: n = 7; D2: n = 6). Two-way ANOVA with Sidak's multiple comparisons test. *: $P < 0.05$.

F. Prolonged time-window recordings of digging associated calcium activity ($\Delta F/F_0$) of M1 and striatal dMSNs (light green) or iMSNs (dark green) from D1R-Cre or D2R-Cre mice.

Data were presented as mean ± SEM.

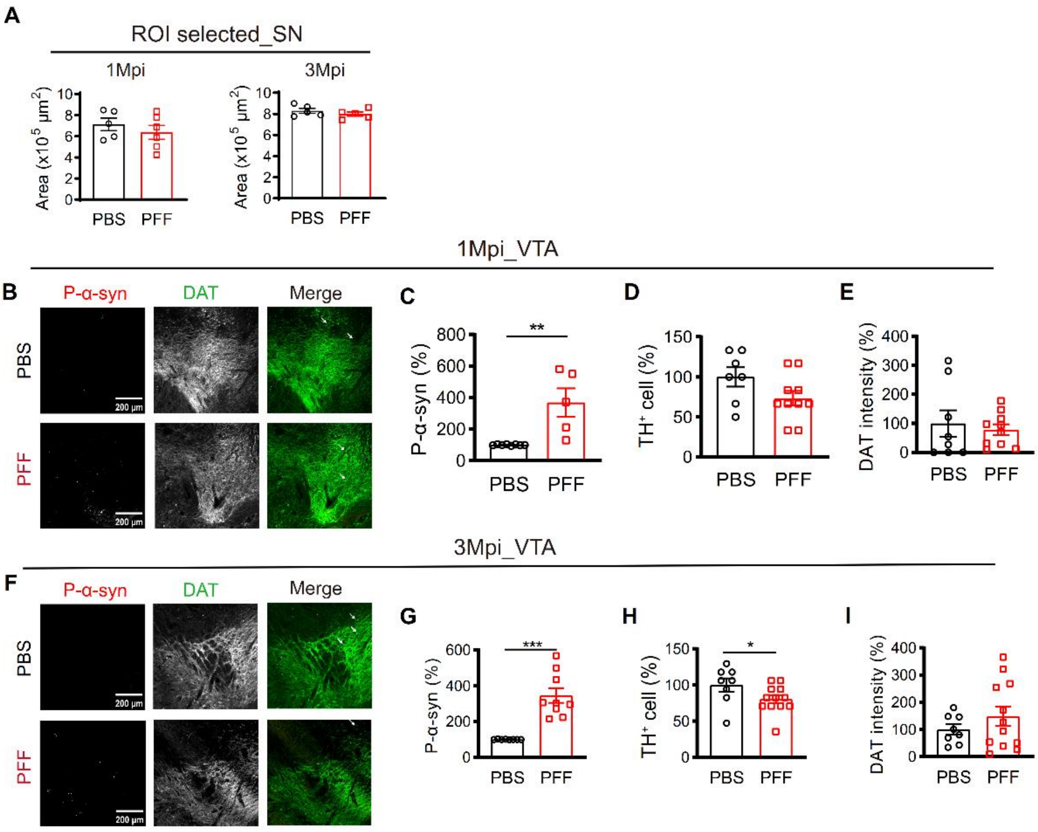

Figure S3. Pathologies in VTA region of PD-like mice. Related to Fig3.

A. Areas of substantia nigra (SN) analyzed in Fig.3K and 3R were comparable between PBS and PFF mice. 1Mpi, PBS: n = 5; PFF: n = 6. 3Mpi, PBS: n = 5; PFF: n = 5.

B-E. Immunofluorescence analyses of pathological hallmarks in VTA regions in PFF- and PBS-injected mice at 1 Mpi. A, Representative immunofluorescence images. Scale bars: 200 μm. (B-D) Comparisons of the p-α-Syn intensity (B, *P* = 0.0016), DA neuron number (C) and DAT intensity (D) in VTA. All data were normalized to PBS control (PBS: 8 views from 2 mice; PFF: 10 views from 3 mice).

F-I. Immunofluorescence analyses of pathological hallmarks in VTA regions in PFF- and PBS-injected mice at 3 Mpi. All data normalized to PBS control. E, Representative immunofluorescence images. Scale bars: 200 μm. (F-H) Comparison of the p-α-Syn intensity (F, *P* < 0.0001), *TH+* neuron number (G, *P* = 0.0368) and DAT intensity (H) in VTA (PBS: 8 views from 2 mice; PFF: 12 views from 2 mice).

Mann-Whitney test, two-tailed. *: P < 0.05. **: P < 0.01. ***: P < 0.001. Data were presented as mean ± SEM.

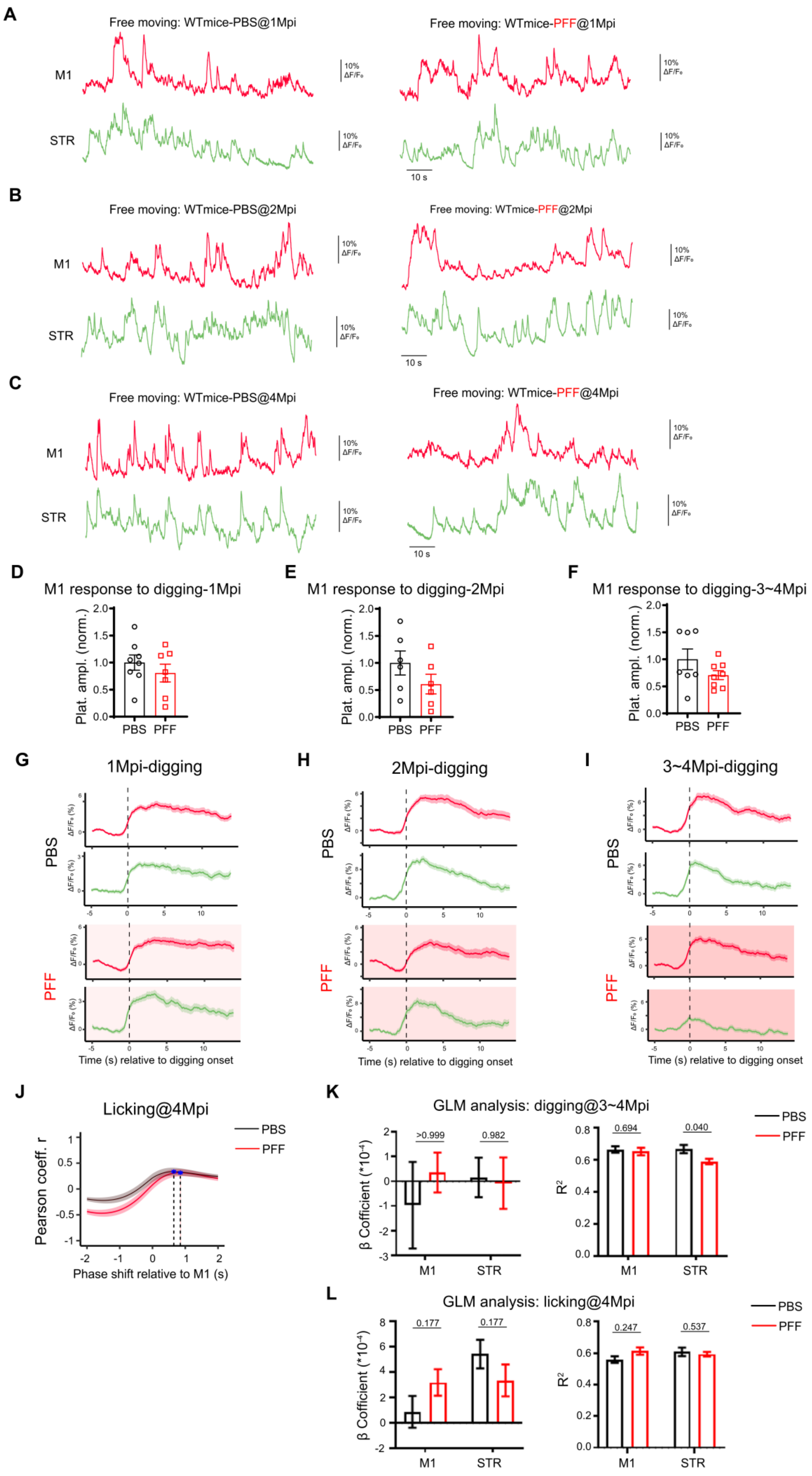
A
Free moving: WTmice-PBS@1Mpi
Free moving: WTmice-PFF@1Mpi
M1
STR
10 s
B
Free moving: WTmice-PBS@2Mpi
Free moving: WTmice-PFF@2Mpi
C
Free moving: WTmice-PBS@4Mpi
Free moving: WTmice-PFF@4Mpi
D
M1 response to digging-1Mpi
E
M1 response to digging-2Mpi
F
M1 response to digging-3~4Mpi
Plat. ampl. (norm.)
PBS
PFF
G
1Mpi-digging
H
2Mpi-digging
I
3~4Mpi-digging
Time (s) relative to digging onset
J
Licking@4Mpi
Pearson coeff. r
Phase shift relative to M1 (s)
K
GLM analysis: digging@3~4Mpi
β Cofficient (*10⁻⁴)
>0.999
0.982
0.694
0.040
R²
L
GLM analysis: licking@4Mpi
0.177
0.177
0.247
0.537

Figure S4. Cortico-striatal decoupling in PFF mice was behavior-specific. Related to Fig4.

A-C. Example temporally aligned Ca^{2+} activities recorded from M1 (red) and STR (green) from a PBS- (left) or PFF-injected mouse (right) during free moving at 1 Mpi (A), 2 Mpi (B), 4 Mpi (C).

D-F. Normalized plateau amplitude of M1 digging-associated Ca^{2+} activities from PBS- and PFF-injected mice at 1 Mpi (D, PBS: n = 8; PFF: n = 7), 2 Mpi (E, PBS: n = 6; PFF: n = 6), 3~4 Mpi (F, PBS: n = 7; PFF: n = 8). Data batch-normalized to the PBS group.

G-I. Prolonged time-window recordings of digging associated calcium activity ($\Delta F/F_0$) of M1 and striatum from PBS or PFF mice at 1 Mpi (G), 2Mpi (H) or 3~4Mpi (**I**).

J. Cross-correlation lag distributions of licking-related calcium signals from PBS- (black) or PFF-injected (red) mice at 4Mpi. PBS: n = 5; PFF: n = 6.

K-L. General linear model (GLM) shows a selective disruption of M1–striatal coupling during digging (K) but not licking (L) behavior in α-synuclein PFF mice. (K) left panel, GLM analysis showing the mean regression coefficient (β) between motion energy and $\Delta F/F_0$ signals in M1 and STR during digging behavior for PBS (black) and α-synuclein PFF (red) mice 3-month post injection. Right panel, the corresponding R^2 values show reduced explanatory power of the GLM fits in PFF mice. (L) During licking behavior, both β coefficients and R^2 values remained largely comparable between PBS and PFF groups (3-4-month post injection).

Data were analyzed using Mann-Whitney test, two-tailed and presented as mean ± SEM.

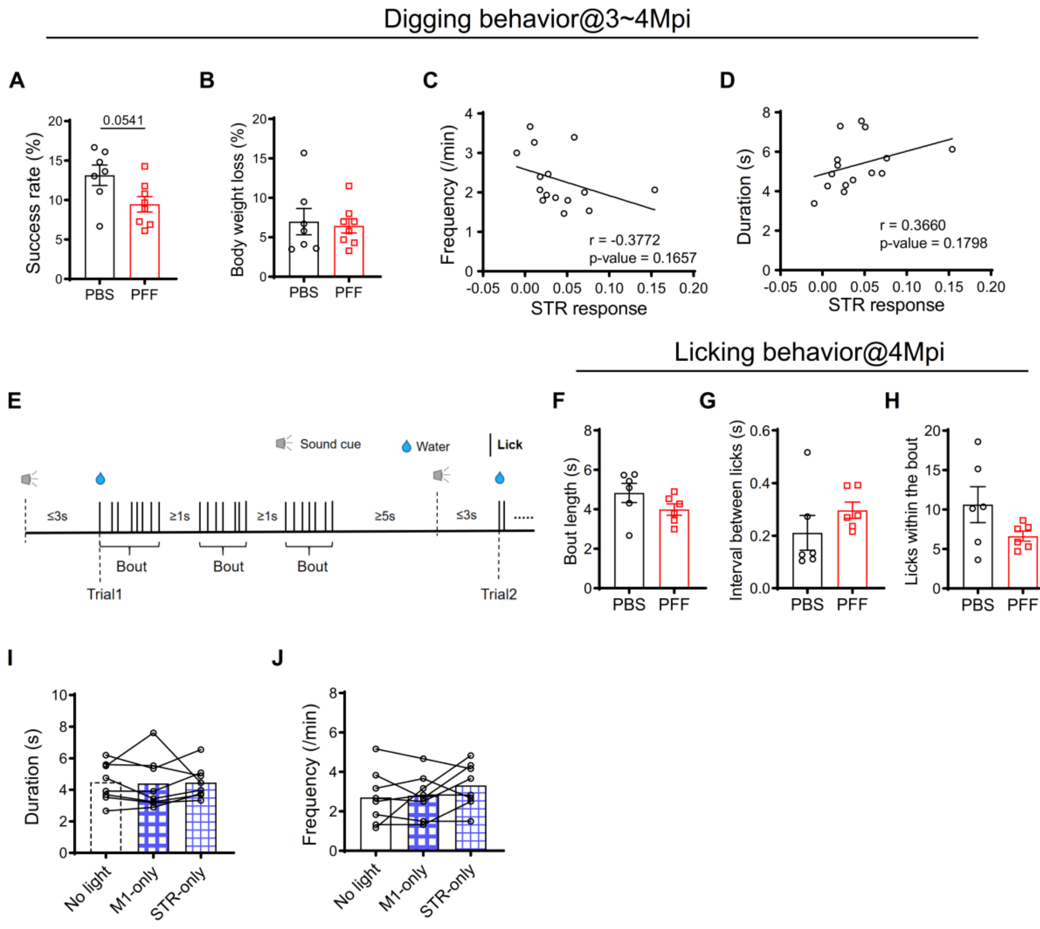

Figure S5. Behavioral phenotypes in PBS- or PFF-injected and ChR2-expressing wild-type mice. Related to Fig5.

A. Success rate of digging (Number of trials retrieving pellets / Number of total trials * 100%).

B. Body weight loss after 24h food deprivation.

C-D. Correlation analysis between STR responses (plateau amplitude, $\Delta F/F_0$) and digging duration (C) or frequencies (D). r, Pearson's correlation coefficient.

E. Paradigm of sound-cued licking test. See details in methods.

F-H. Characterization of licking behavior. (F) Average bout duration. (G) Average lick interval within a bout. (H) Average lick counts per bout.

I. Diagrams depicting the optogenetic stimulation protocols: three 6-minute epochs: no light stimulation, stimulation of M1 alone (M1-only) and stimulation of STR alone (STR-only).

J. Optogenetic stimulation of M1 or STR alone did not affect digging duration (K_1) or total digging events (K_2). One-way ANOVA followed by Šídák's multiple comparisons test.

Data were analyzed using two-tailed Mann-Whitney test unless noted; data were presented as

mean ± SEM.

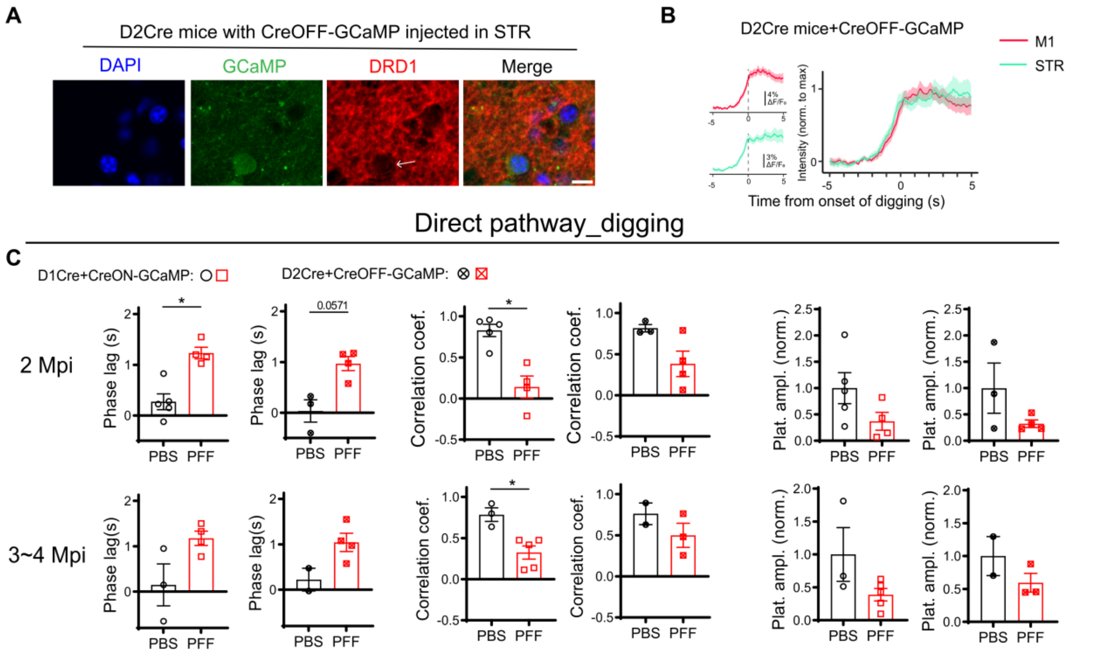

Figure S6. CreON and CreOFF systems equally enabled detection of the abnormal M1-dMSNs coupling associated with digging in PFF mice. Related to Fig6.

A. Representative immunofluorescence images showing the co-localization of GCaMP and D1R immunoreactivity (arrow) from a D2R-Cre mouse expressing Cre-OFF GCaMP. Scale bar: 10 μm

B. $\Delta F/F_0$ traces of M1 (red) and STR dMSNs (aqua) Ca^{2+} activities temporally aligned to the initiation of digging in 3 D2R-Cre mice injected with CreOFF-GCaMP. The average $\Delta F/F_0$ waveform of M1 (Top left panels) and STR (Bottom left panels) were presented, and the waveform for both regions were overlaid after normalizing each channel to its maximum value (panels on the right).

C. M1-dMSNs correlation data collected from D1R-Cre mice using Cre-on GCaMP (Open symbols) and D2R-Cre mice with Cre-off GCaMP (Cross-filled symbols). 2 Mpi, PBS: n = 8; PFF: n = 8. 3~4 Mpi, PBS: n = 5; PFF: n = 8.

Data were analyzed using Mann-Whitney test, two-tailed and presented as mean ± SEM. *: $P < 0.05$. Each point represents a single mouse.

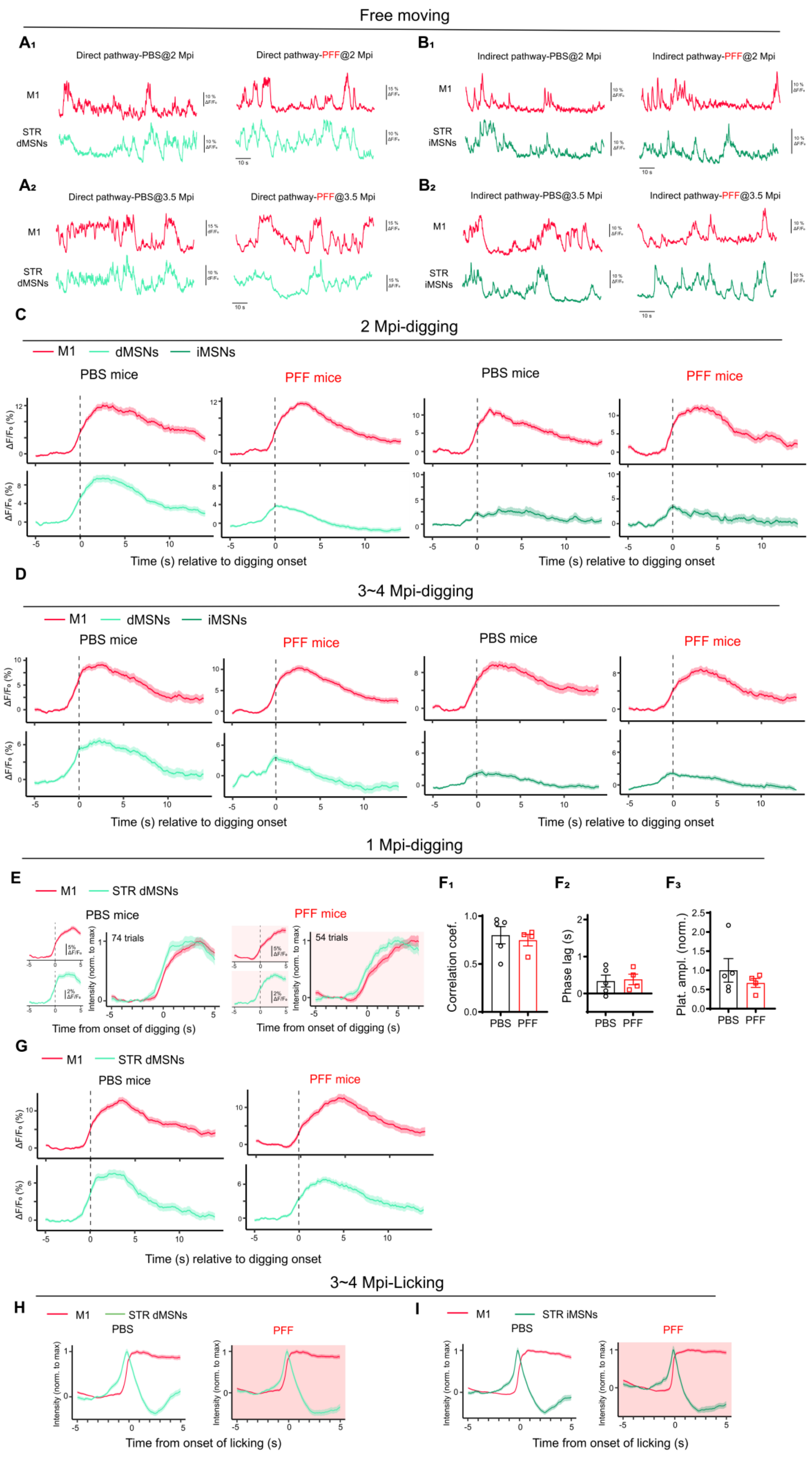

Free moving
A1
Direct pathway-PBS@2 Mpi
Direct pathway-PFF@2 Mpi
B1
Indirect pathway-PBS@2 Mpi
Indirect pathway-PFF@2 Mpi
M1
STR dMSNs
STR iMSNs
A2
Direct pathway-PBS@3.5 Mpi
Direct pathway-PFF@3.5 Mpi
B2
Indirect pathway-PBS@3.5 Mpi
Indirect pathway-PFF@3.5 Mpi
C
2 Mpi-digging
M1
dMSNs
iMSNs
PBS mice
PFF mice
ΔF/F₀ (%)
Time (s) relative to digging onset
D
3~4 Mpi-digging
1 Mpi-digging
E
M1
STR dMSNs
74 trials
54 trials
Intensity (norm. to max)
Time from onset of digging (s)
F1
Correlation coef.
F2
Phase lag (s)
F3
Plat. ampl. (norm.)
PBS
PFF
G
3~4 Mpi-Licking
H
I
STR iMSNs
Time from onset of licking (s)

Figure S7. Ca^{2+} activities traces recorded from M1 and STR in behavior- and cell type-specific manner. Related to Fig6.

A. Example temporally aligned Ca^{2+} activities traces recorded from M1 (red) and STR dMSNs (aqua) of PBS- (left) or PFF-injected mice (right) during free moving at 2 Mpi (A_1) and 3.5 Mpi (A_2).

B. Example temporally aligned Ca^{2+} activities traces recorded from M1 (red) and STR iMSNs (dark green) of PBS- (left) or PFF-injected mice (right) during free moving at 2 Mpi (B_1) and 3.5 Mpi (B_2).

C. Prolonged time-window recordings of digging associated calcium activity ($\Delta F/F_0$) of M1 and striatal dMSNs (light green) or iMSNs (dark green) from PBS or PFF mice at 2 Mpi.

D. Prolonged time-window recordings of digging associated calcium activity ($\Delta F/F_0$) of M1 and striatal dMSNs (light green) or iMSNs (dark green) from PBS or PFF mice at 3~4 Mpi.

E-G. Dual-site fiber photometry imaging of M1 and striatal dMSNs in PFF- or PBS-injected mice at 1 Mpi (PBS: 74 trials from 5 mice; PFF: 54 trials from 4 mice) during digging. (E) Normalized $\Delta F/F_0$ traces of M1 (red) and striatal dMSNs (green) Ca^{2+} activities temporally aligned to the initiation of digging at 1 Mpi in mice injected with either PBS (panels with white background) or PFF (panels in pink background). The average $\Delta F/F_0$ waveform of M1 (Top left panels) and STR (Bottom left panels) were presented, and the waveform for both regions were overlaid after normalizing each channel to its maximum value (panels on the right). (F) Quantitative analyses of the M1-dMSNs correlation and normalized plateau amplitudes of dMSN response at 1 Mpi. (G) Prolonged time-window recordings of digging associated calcium activity ($\Delta F/F_0$) of M1 and striatal dMSNs (light green) from PBS or PFF mice at 1 Mpi.

H-I. Normalized $\Delta F/F_0$ traces of M1 (red) and STR dMSNs (H, PBS: n = 131 trials from 3 mice, PFF:133 trials from 3 mice) or iMSNs (I, 124 trials from 3 mice, PFF:162 trials from 4 mice) licking-associated Ca^{2+} activities from PBS- and PFF-injected mice at 3~4 Mpi.

Each point represents a single mouse. Data were analyzed using Mann-Whitney test, two-tailed and presented as mean ± SEM.

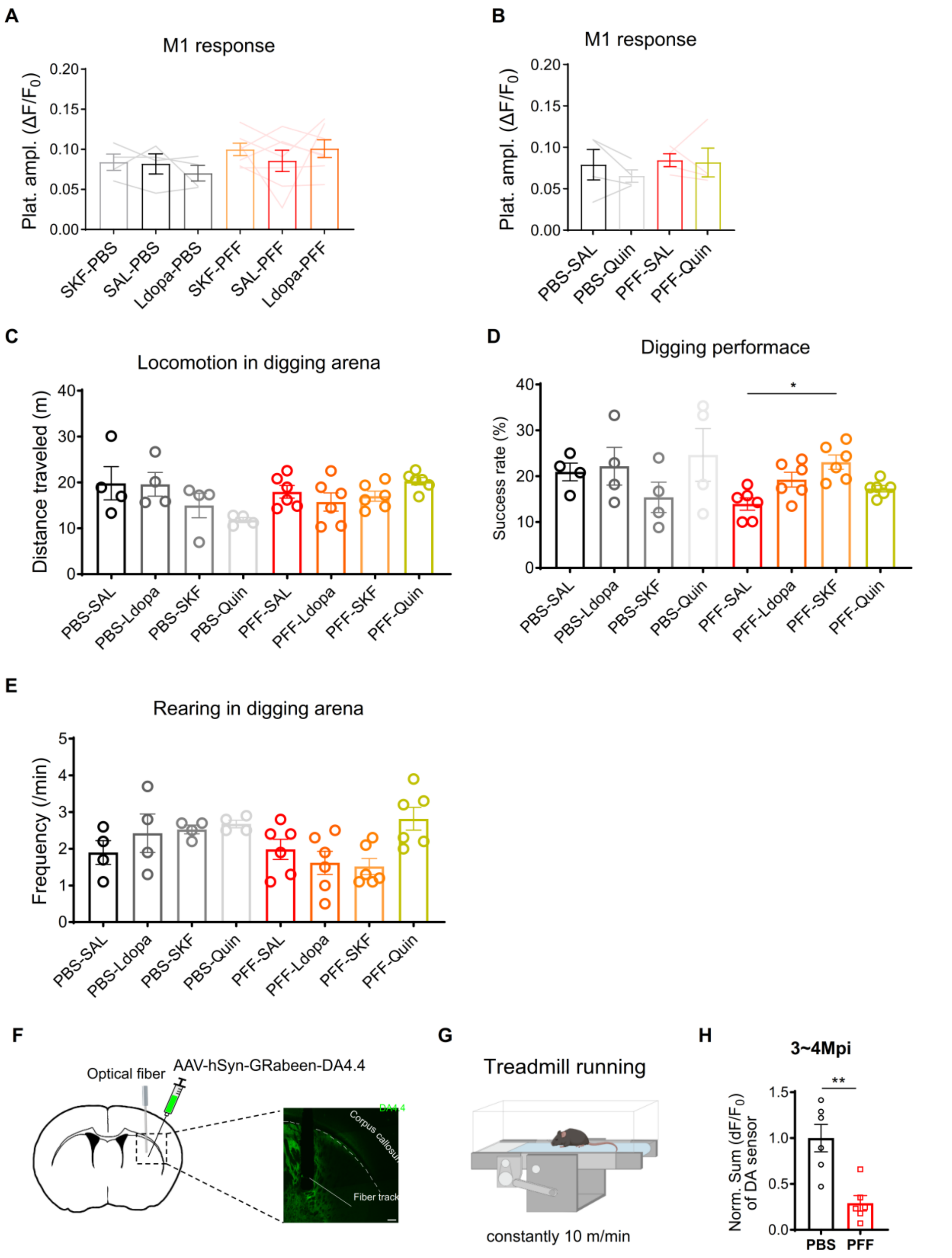

Figure S8. Effects of dopaminergic drugs on M1 responses and digging behavior. Related to Fig7.

A-B. Plateau amplitudes of digging-associated M1 Ca^{2+} activities of PBS- and PFF-injected mice after administration of different drugs. Wilcoxon matched-pairs test (B). A, PBS: n = 4; PFF: n = 7. B, PBS: n = 4; PFF: n = 4.

C. Travel distances during the first 10 minutes after administration of different drugs.

D. Effects of different drugs on digging success rates. (One-way ANOVA, $F_{(2.031, 10.16)} = 6.532$. SAL-PFF *vs.* SKF-PFF, *P* = 0.0478, Dunnett's multiple comparisons test). PBS: n = 4; PFF: n = 7.

E. Frequencies of rearing during the first 10 minutes after administration of different drugs.

F. Schematic diagram showing the experimental design for assessing striatal extracellular dopamine (DA) level. DA4.4 is a florescence-based DA sensor. Right panel, anatomical verification of virus expression and optic fiber localization. Scale bar: 200 μm.

G. Schematic diagram for treadmill running test.

H. Normalized sum activity of DA sensor during treadmill running from PBS- and PFF-injected mice at 3~4 Mpi (*P* = 0.0043). Mann-Whitney test, two-tailed. PBS: n = 6; PFF: n = 6.

One-way ANOVA followed by Dunnett's multiple comparisons test, unless otherwise noted. *: $P < 0.05$. Each point represents a single mouse. Data were presented as mean ± SEM.

Table of materials

Antibodies	Host species	Company	Identifier	Dilution
Phospho-α-syn (S129)	rabbit	Abcam	ab51253	1:250
DAT	rat	Abcam	ab5990	1:500
Alexa Fluor 568	goat (anti-rabbit)	Abcam	ab175471	1:1000
Alexa Fluor 647	goat (anti-rat)	Abcam	ab150159	1:150
ChAT	rabbit	Proteintech	20747-1-AP	1:400
DRD1	rabbit	Proteintech	17934-1-AP	1:200
DRD2	rabbit	Proteintech	55084-1-AP	1:200
Alexa Fluor 647	goat (anti-rabbit)	Thermo Fisher	A21245	1:1000

Chemicals	Product/chemical name	Company	CAS number	Note
SKF-81297	6-Chloro-PB hydrobromide	Sigma-Aldrich	71636-61-8	D1R agonist
Quinpirole	Quinpirole Hydrochloride	Sigma-Aldrich	85798-08-9	D2R agonist
L-dopa	Levodopa	Sigma-Aldrich	59-92-7	Precursor of dopamine
Benserazide	Benserazide hydrochloride	Sigma-Aldrich	14919-77-8	Combined with L-dopa
